# NEAT1 as a competing endogenous RNA in tumorigenesis of various cancers: Role, mechanism and therapeutic potential

**DOI:** 10.7150/ijbs.62728

**Published:** 2021-08-03

**Authors:** Kun Li, Tongyue Yao, Yu Zhang, Wen Li, Ziqiang Wang

**Affiliations:** 1 Department of Nuclear Medicine, The First Affiliated Hospital of Shandong First Medical University & Shandong Provincial Qianfoshan Hospital, Jinan 250014, China.; 2 Biomedical Sciences College & Shandong Medicinal Biotechnology Centre, Shandong First Medical University & Shandong Academy of Medical Sciences, Jinan 250062, China.

**Keywords:** NEAT1, competing endogenous RNA, long non-coding RNA, microRNA, cancer, therapeutic target

## Abstract

The nuclear paraspeckle assembly transcript 1 (NEAT1) is a long non-coding RNA (lncRNA) that is upregulated in a variety of human cancer types. Increasing evidence has shown that the elevation of NEAT1 in cancer cells promotes cell growth, migration, and invasion and inhibits cell apoptosis. It is also known that lncRNAs act as a competing endogenous RNA (ceRNA) by sponging microRNAs (miRNAs) to alter the expression levels of their target genes in the development of cancers. Therefore, it is important to understand the molecular mechanisms underlying this observation. In this review, specific emphasis was placed on NEAT1's role in tumor development. We also summarize and discuss the feedback roles of NEAT1/miRNA/target network in the progression of various cancers. As our understanding of the role of NEAT1 during tumorigenesis improves, its therapeutic potential as a biomarker and/or target for cancer also becomes clearer.

## Introduction

Nuclear paraspeckle assembly transcript 1 (NEAT1) is a long non-coding RNA (lncRNA) located in nuclear paraspeckles. It functions as a frame for paraspeckle formation by associating with the paraspeckle proteins, paraspeckle component 1 (PSPC1), splicing factor proline/glutamine rich (SFPQ), and 54 kDa nuclear RNA- and DNA-binding protein (p54nrb) 1,2. Since the discovery of NEAT1 in 2007, many of its biological functions have been reported, including regulation of cell differentiation 3,4, immune response 5, and organ development 6,7; NEAT1 also participates in the progression of a variety of disorders, such as cancer 8,9, metabolic diseases 10,11, and immunological diseases 12. In addition, our previous studies revealed that NEAT1 is also involved in herpes simplex virus-1 (HSV-1) replication and the development of Alzheimer's disease (AD) by epigenetically regulating the expression of HSV-1 viral genes and endocytosis-related genes, respectively 13,14. The key role of NEAT1 is to mediate gene expression through complex mechanisms. NEAT1 regulates target genes by recruiting and/or sequestering transcriptional factors and regulators to and from promoters and transcripts of target genes, thereby influencing their transcription, splicing, RNA stability, and translation 15.

There is growing evidence that lncRNAs can act as competing endogenous RNAs (ceRNAs) by sponging microRNAs (miRNAs) to alter the expression levels of their target genes in the development of human diseases 16,17. During tumorigenesis and cancer progression, many oncogenic lncRNAs exhibit dysregulated expression, which promotes the development of cancer and is associated with poor overall survival. This occurs through lncRNA's enhancing of cancer cell proliferation, migration, invasion, and apoptosis inhibition. Researchers discovered that lncRNAs regulate the expression of tumor-related genes by interacting with lncRNA-specific miRNAs, thereby preventing the degradation of tumor-related gene transcripts and promoting their translation 18. These findings suggest that lncRNA-mediated ceRNA networks have great potential as biomarkers and therapeutic targets for cancer.

In this review, we discuss the roles of NEAT1 in the progression of different types of tumors by describing a universal regulatory pattern of NEAT1 in tumor-related gene expression. We have focused on the function of NEAT1 as a ceRNA to upregulate these gene expression levels through sponging miRNAs. This gene upregulation results in the promotion of tumor cell proliferation, migration, invasion, epithelial-mesenchymal transition (EMT), and cell apoptosis inhibition. We also discuss the potential clinical applications of NEAT1-mediated ceRNA networks in overcoming chemo- and radioresistance in cancer treatment.

## NEAT1 role in the tumorigenesis in respiratory system tumors

In this section, we summarize the roles of the NEAT1/miRNA/target axis in respiratory system tumors, including nasopharyngeal carcinoma, sinonasal squamous cell carcinoma, laryngeal squamous cell cancer, and non-small cell lung cancer (Table 1).

### Nasopharyngeal carcinoma

Nasopharyngeal carcinoma (NPC), a common head and neck cancer originating from the nasopharynx epithelium, is a leading cause of cancer-related deaths worldwide 19. In a study of the underlying mechanism of NEAT1 in NPC progression, NEAT1 expression was found to be upregulated in NPC tissues and cells, and NEAT1 knockdown resulted in an inhibition of tumor cell proliferation, migration, invasion, and EMT by blocking Wnt/β-catenin signaling, a trigger for tumorigenesis 20, via targeting miR-34a-5p 21.

NF-κB signaling is another pathway that is influenced by NEAT1 in NPC progression. Cheng et al. reported that miR-124 inhibits NPC cell proliferation and promotes cell apoptosis by binding to and repressing the expression of NEAT1 and NF-κB, suggesting that NEAT1 functions as a potential ceRNA for NF-κB expression 22.

### Sinonasal squamous cell carcinoma

Sinonasal squamous cell carcinoma (SNSCC) is the most common type of sinonasal malignancy, an aggressive tumor type characterized by late discovery and rapid progression 23,24. A study on the molecular mechanisms of SNSCC development revealed that the expression of lncRNA NEAT1 and vascular endothelial growth factor A (VEGFA) were both upregulated in SNSCC tissues and cells, which resulted in a promotion of SNSCC cell viability and a reduction in SNSCC cell apoptosis. Moreover, upregulation of miR-195-5p in SNSCC cells decreased cell viability by directly binding with NEAT1 and VEGFA and decreasing their expression levels, suggesting an important role of the NEAT1/miR-195-5p/VEGFA axis in SNSCC progression 25.

### Laryngeal squamous cell cancer

Laryngeal squamous cell carcinoma (LSCC) is the most common malignant tumor occurring in the head and neck, and it also is the leading cause of cancer-related deaths in this category 26. Wang et al. 27 studied the role of NEAT1 in human LSCC progression, and the authors found that NEAT1 expression was significantly induced in LSCC with a positive relationship with grade, lymph node metastasis, and clinical stages. Moreover, NEAT1 was shown to promote LSCC cell proliferation and invasion and inhibit LSCC cell apoptosis and cell cycle arrest at the G1 phase. Further investigation of the molecular mechanism demonstrated that NEAT1 sponges miR-107 to upregulate the expression of cyclin-dependent kinase 6 (CDK6), a member of the CDK family that significantly correlates with head and neck squamous cell carcinoma progression 28.

### Non-small cell lung cancer

Lung cancer remains one of the most prevalent malignant tumors and the leading cause of cancer-related deaths all over the world 29. The most prevalent form of lung cancer (~80%) is the non-small cell lung cancer (NSCLC). Sun et al. 30 reported that high NEAT1 expression in NSCLC is related to a short overall survival of patients with NSCLC by promoting cancer cell growth and metastasis. Further investigation revealed that NEAT1 functions as a ceRNA for E2F3, a core oncogene in promoting NSCLC progression, by sponging hsa-miR-377-3p and antagonizing its functions of binding with E2F3 to repress E2F3 expression. Furthermore, NEAT1 was found to promote growth, migration, and invasion of NSCLC by sponging miR-98-5p, miR-101-3p, and miR-376b-3p to upregulate mitogen-activated protein kinase 6 (MAPK6) 31, SRY-box transcription factor 9 (SOX9) 32, and sulfatase 1 (SULF1) 33, respectively. These targets play a vital role in cancer progression 34-36.

Lung adenocarcinoma (LUAD) is a histopathological subtype of NSCLC that accounts for nearly 40% of lung cancer cases 37,38. Xiong et al. 39 showed that NEAT1 accelerated LUAD cell proliferation, invasion, and migration and inhibited cell apoptosis by upregulating the expression of upstream stimulating factor 1 (USF1), a basic helix-loop-helix-zipper transcription factor that promotes lung adenocarcinoma progression 40, by sponging miR-193a-3p.

## NEAT1 role in the tumorigenesis in digestive system tumors

In this section, we summarize the roles of the NEAT1/miRNA/target axis in digestive system tumors, including oral squamous cell carcinoma, esophageal squamous cell carcinoma, gastric cancer, hepatocellular carcinoma, and colorectal cancer (Table 2).

### Oral squamous cell carcinoma

Oral squamous cell carcinoma (OSCC) is the most prevalent type of head and neck squamous cell carcinoma (HNSCC), the sixth most common cancer worldwide in 2018 41,42. In a study to determine the function and mechanism of lncRNA NEAT1 in OSCC, NEAT1 expression was found to be significantly upregulated in OSCC cells and tissues. This high expression of NEAT1 was positively correlated with advanced TNM stage (a system used to classify **T**umor size, **N**ode location, and **M**etastasis status) and poor survival of patients by promoting tumor cells proliferation and invasion, and inhibiting cell cycle arrest at the G0/G1 phase and apoptosis. It is proposed that NEAT1 could positively regulate the expression of the regulator of G protein signaling 20 (RGS20), an accelerator for the proliferation and migration of cancer cells 43,44, by interacting with miR-365 to suppress the repressive effects of miR-365 on the expression of RGS20 45.

### Esophageal squamous cell carcinoma

Esophageal squamous cell carcinoma (ESCC) is the predominant histological type of esophageal cancer and is one of the most common and leading aggressive malignancies, with a five-year survival rate of less than 10% 46. A study investigating the molecular mechanism of the NEAT1 regulatory network in ESCC progression revealed that the expression of NEAT1 and C-terminal-binding protein 2 (CTBP2) was upregulated, while expression of miR-129 was downregulated in ESCC cells. Further studies validated that miR-129 could target NEAT1 and CTBP2 to decrease their expression levels. In addition, cellular function investigation confirmed that either NEAT1 knockdown, CTBP2 knockdown, or miR-129 upregulation resulted in an inhibition of ESCC cell viability and invasion, suggesting a NEAT1/miR-129/CTBP2 regulatory network in ESCC progression 47.

### Gastric cancer

Gastric cancer (GC) remains the third leading cause of cancer-related deaths all over the world and is the most common type of digestive malignancies 48-50. In addition, most patients with GC exhibit malignant metastasis with poor overall survival 51,52.

Tan et al. 53 explored the detailed roles and molecular mechanisms of NEAT1 in GC progression. The authors found that the expression of NEAT1 and signal transducer and activator of transcription 3 (STAT3) were significantly upregulated in human GC cells, while expression of miR-506 was downregulated. NEAT1 and STAT3 are two targets of miR-506. Moreover, NEAT1 knockdown repressed GC cell growth, migration, and invasion by decreasing the expression level of STAT3 via miR-506 upregulation.

In addition, other NEAT1 sponging-miRNAs and targets of these miRNAs that play roles in GC progression have been discovered. For example, the NEAT1/miR-497-5p/phosphoinositide-3-kinase regulatory subunit 1 (PIK3R1) axis promotes GC cell proliferation and inhibits GC cell apoptosis 54; the NEAT1/miR-335-5p/rho associated coiled-coil containing protein kinase 1 (ROCK1) axis promotes GC cell proliferation, migration, and invasion 55; the NEAT1/miR-103a/STAM binding protein like 1 (STAMBPL1) axis promotes GC cell proliferation and cell invasion 56; NEAT1/miR-365a-3p/ATP binding cassette subfamily C member 4 (ABCC4) axis promotes GC cell proliferation, colony formation, invasion, and cell cycle progression 57.

### Hepatocellular carcinoma

Hepatocellular carcinoma (HCC) is the fifth most common cancer in men and seventh in women and is the second most common cause of cancer-related deaths worldwide 58,59. Increasing evidence has demonstrated that NEAT1 expression is induced and NEAT1 upregulation promotes HCC progression. Molecular mechanism investigations have shown that NEAT1 promotes HCC cell proliferation, migration, and invasion by upregulating the expression of calponin 2 (CNN2), transforming growth factor-β1 (TGF-β1), and STAT3 by targeting miR-296-5p, miR-139-5p, and miR-485, respectively 60-62.

### Colorectal cancer

Colorectal cancer (CRC) remains the second most common cause of cancer-related deaths in the United States 63. He et al. reported that NEAT1 knockdown inhibited colon cancer cell proliferation, cell cycle, cell migration/invasion, and promoted colon cancer cell apoptosis by repressing the expression of CDK6 via interaction with miR-495-3p 64. CDK6 is a member of the CDK family whose dysregulation in cancers results in continued proliferation and unscheduled cell cycle 65. Moreover, the NEAT1/miR-185-5p/insulin-like growth factor 2 (IGF2) axis is another pathway that induces the invasion and migration of colon cancer 66.

In addition, NEAT1 upregulation in CRC was significantly correlated with poor TNM staging, survival, and tumor recurrence in patients with CRC. By upregulating the expression of sirtuin-1 (SIRT1) via miR-34a 67, glial cell-derived neurotrophic factor (GDNF) via miR-196a-5p 68, and VEGFA via miR-205-5p 69, NEAT1 enhanced CRC cell proliferation, colony formation, and invasive potential. By upregulating the expression of interleukin 17 receptor D (IL17RD) via miR-193a 70, solute carrier family 38 member 1 (SLC38A1) via miR-138 71, KRAS via miR-193a-3p 72, and centrosomal protein 55 (CEP55) via miR-195-5p 73, NEAT1 promotes CRC cell proliferation, migration, and invasion and inhibits apoptosis.

## NEAT1 role in the tumorigenesis in reproductive system tumors

In this section, we summarize the roles of the NEAT1/miRNA/target axis in reproductive system tumors, including breast cancer, ovarian cancer, cervical cancer, endometrial carcinoma, and prostate cancer (Table 3).

### Breast cancer

Breast cancer (BC) remains the leading cause of cancer death in women and occurs in the epithelial tissue of the mammary gland 74. Researchers found that NEAT1 overexpression in BC was correlated with poor prognosis of patients and the feedback loop of NEAT1/miR-107/carnitine palmitoyltransferase 1A (CPT1A) 75, NEAT1/miR-124/STAT3 76, NEAT1/miR-448/zinc finger E-box binding homeobox 1 (ZEB1) 77, and NEAT1/miR-101/enhancer of zeste homolog 2 (EZH2) 78, which promotes BC cell proliferation, migration, invasion, and cell cycle progression. In addition, NEAT1 upregulation in BC enhances EMT and inhibits cell apoptosis by sponging miR-410-3p to upregulate the expression of cyclin D1 (CCND1) 79 and sponging miR-138-5p to upregulate the expression of zinc finger protein X-linked (ZFX) 80.

### Ovarian cancer

Ovarian cancer (OC) is another leading cause of cancer-related deaths in the female population worldwide 81. Ding et al. 82 reported that NEAT1 overexpression in OC promoted proliferation and inhibited apoptosis of OC cells by negatively regulating miR-34a-5p expression and positively regulating B-cell lymphoma-2 (BCL2), a target of miR-34a-5p. Moreover, NEAT1 enhanced the metastasis of OC cells by upregulating the expression of ROCK1 by sponging miR-382-3p 83.

In addition to the promotion of OC cell proliferation, migration, and invasion, NEAT1 was shown to inhibit OC cell apoptosis by upregulating the expression of basic leucine zipper and W2 domain‑containing protein 1 (BZW1) via interaction with miR-4500 84. NEAT1 also enhanced EMT of OC cells by upregulating the expression of tight junction protein 3 (TJP3) via interaction with miR-1321 85.

### Cervical cancer

Cervical cancer (CC) remains the second most common and serious malignant tumor among women all over the world 86. Xie et al. studied the role of NEAT1 in CC progression, and the authors reported that NEAT1 upregulation in CC tissue enhanced CC cell proliferation and migration 87. Mechanistically, NEAT1 functions as a ceRNA to bind miR-9-5p and increase the expression level of POU class 2 homeobox 1 (POU2F1), a target of miR-9-5p. Moreover, overexpression of NEAT1 could inhibit CC cell apoptosis and EMT by targeting miR-133a, thereby increasing the expression of SRY-box transcription factor 4 (SOX4), an important epigenetic regulator in tumorigenesis 88, 89, and targeting miR-361 to increase expression of the 90-kDa heat shock proteins (HSP90s), an essential factor contributing to the tumor metastatic phenotype 90,91.

### Endometrial carcinoma

Endometrial carcinoma (EC) is a commonly diagnosed gynecological cancer worldwide, and its incidence is increasing 92. Researchers investigated the function and mechanism of lncRNA NEAT1 in EC progression, and they found that NEAT1 promotes EC cell proliferation, migration, and invasion by sponging miR-214-3p and miR-144-3p to upregulate the expression of high mobility group AT-hook 1 (HMGA1) 93 and EZH2 94, respectively.

### Prostate cancer

Prostate cancer (PCa) is the second most common tumor and the fifth leading cause of cancer-related deaths among men 42. Guo et al. reported that NEAT1 expression was significantly upregulated in PCa tissues and PCa cell lines, and NEAT1 knockdown inhibited the growth and invasion of PCa cells 95. Mechanistically, NEAT1 upregulates the expression of high mobility group AT-hook 2 (HMGA2), an important transcription factor for genes that modulate cell cycle process, DNA damage, apoptosis, and EMT 96, by binding miR-98-5p and decreasing the expression level of miR-98-5p.

### NEAT1 in the tumorigenesis in circulatory system tumors

In this section, we summarize and discuss the role of the NEAT1/miRNA/target axis in circulatory system tumors, including hemangioma, acute myeloid leukemia, T-cell acute lymphoblastic leukemia, diffuse large B-cell lymphoma, Hodgkin's lymphoma, and multiple myeloma (Table 4).

### Hemangioma

Hemangioma (HA) is one of the most common benign vascular neoplasms of infancy due to the abnormal proliferation of hemangioma endothelial cells (HemECs) 97. Yu et al. studied the roles and molecular mechanisms of NEAT1 in HA progression; the authors found that NEAT1 expression is increased in hemangiomas and depletion of NEAT1 results in the inhibition of HemEC proliferation, migration, and invasion 98. Investigation of the mechanism revealed that NEAT1 upregulated the expression of HIF1α by sponging miR-33a-5p, thus activating NF-κB signaling, a critical pathway for tumorigenesis 99. In addition, NEAT1 was found to inhibit the apoptosis of HemECs, thereby contributing to HA progression, by interacting with miR-361-5p to upregulate the expression of VEGFA, an essential factor in promoting cancer progression by increasing the proliferation and migration of cancer cells 100,101.

### Acute myeloid leukemia

Acute myeloid leukemia (AML) is a representative hematologic malignancy characterized by an abnormal abundance of aberrantly differentiated myeloid cells in the bone marrow 102. Researchers investigated the regulatory influence of the NEAT1/miRNA/target axis in AML progression; they found that NEAT1 expression was downregulated in AML cells and that overexpression of NEAT1 inhibited cell proliferation, migration, and invasion, decreased the number of cells in the G2/M phase, and significantly induced cell apoptosis through the NEAT1/miR-23a-3p/structural maintenance of chromosomes 1A (SMC1A) axis 103 and NEAT1/miR-338-3p/CREB3 regulatory factor (CREBRF) axis 104.

### T-cell acute lymphoblastic leukemia

T-cell acute lymphoblastic leukemia (T-ALL) is an aggressive leukemia originating from T-lymphocytes in the bone marrow. Patients show symptoms of weakness, enlarged lymph nodes, fatigue, and weight loss 105. Luo et al. studied the regulatory mechanism of NEAT1 in the process of T-ALL 106. The authors found that NEAT1 expression levels were markedly increased in T-ALL cells. NEAT1 promotes the proliferation of T-ALL cells by upregulating the expression of NOTCH1, a driving oncogene that induces the development of pre-T cells to leukemia 107, by sponging miR-146b-5p and decreasing its expression level.

### Diffuse large B-cell lymphoma

Diffuse large B-cell lymphoma (DLBCL) is the most common subtype of non-Hodgkin lymphoma, and it is typically considered an aggressive lymphoma 108. A study to investigate the underlying mechanism of NEAT1 in DLBCL progression found that NEAT1 transcriptionally regulated by MYC was upregulated in DLBCL tissues and cell lines, and NEAT1 knockdown resulted in the inhibition of DLBCL cell proliferation and a promotion of DLBCL cell apoptosis. Mechanistically, NEAT1 functions as a ceRNA to target miR-34b-5p and, thus, increases the expression level of the GLI family zinc finger 1 (GLI1), an oncogene that contributes to cell survival of DLBCL 109, 110.

### Hodgkin's lymphoma

Hodgkin's lymphoma (HL) is the most common malignant lymphoma originating in the lymphoid hematopoietic system, especially in young adults 111. Fan et al. 112 showed that NEAT1 expression was significantly enhanced in HL tissues and cell lines, and NEAT1 downregulation resulted in inhibition of HL cell proliferation and invasion through the downregulation of doublecortin-like kinase 1 (DCLK1), an accelerator in tumor cell invasion, metastasis, and EMT 113, via interaction with miR-448.

### Multiple myeloma

Multiple myeloma (MM) is one of the most common hematological malignancies characterized by aberrant proliferation of plasma cells and secretion of monoclonal immunoglobulin proteins 114. Gao et al. 115 reported that NEAT1 upregulation in MM patients promoted M2 macrophage polarization, a contributor to tumor progression that promotes angiogenesis to support tumor growth 116, by upregulating the expression and release of B7-H3 and then activating JAK2/STAT3 signaling via direct targeting of miR-214.

## NEAT1 in the tumorigenesis in nervous system tumors

In this section, we summarize and discuss the role of the NEAT1/miRNA/target axis in nervous system tumors, including glioma, retinoblastoma, and neuroblastoma (Table 5).

### Glioma

Gliomas are the most common and aggressive tumors of the central nervous system and characterized by extremely poor prognosis outcomes 117. Researchers studied the molecular mechanisms underlying gliomas. They observed that NEAT1 was upregulated in glioma tissues and cell lines, and this upregulation contributed to glioma progression by inducing glioma cell survival, promoting cell proliferation, migration, and invasion by sponging miR-107 118, miR-132 119, and miR-449b-5 120 to elevate the expression levels of cyclin-dependent kinase 14 (CDK14), SRY-box transcription factor 2 (SOX2), and c-Met, respectively. In addition, NEAT1/miR-152-3p/chaperonin containing the TCP1 subunit 6A (CCT6A) axis 121 and NEAT1/miR-139-5p/CDK6 axis 122 were shown to inhibit cell apoptosis, while the NEAT1/miR-185-5p/DNA methyltransferase 1 (DNMT1) axis 123 promoted EMT in glioma cells and inhibited cell apoptosis.

### Retinoblastoma

Retinoblastoma (RB) is an aggressive retinal cancer that is initiated in response to biallelic loss of the tumor suppressor gene RB1 in almost all cases and develops after additional genetic/epigenetic alterations 124. A study on the role of NEAT1 in RB progression revealed that NEAT1 expression levels were elevated in RB tissues and cells; NEAT1 knockdown significantly inhibited RB cell proliferation and migration and promoted cell apoptosis by competitively binding with miR-204 to regulate the expression of C-X-C chemokine receptor type 4 (CXCR4) 125.

### Neuroblastoma

Neuroblastoma (NB) is the most common pediatric solid tumor that arises in the sympathetic nervous system. NB accounts for 7%-8% of childhood malignancies and ~15% of childhood cancer-related deaths 126. Yang et al. explored the mechanism of NEAT1 in NB progression. The authors observed that NEAT1 expression was induced in neuroblastoma cell lines, and overexpression of NEAT1 resulted in an increase in NB cell proliferation and a decrease in cell apoptosis through upregulating the expression of Janus kinase 1 (JAK1) and STAT3 by sponging miR-326 127.

## NEAT1 role in the tumorigenesis in endocrine system tumors

In this section, we summarize and discuss the role of the NEAT1/miRNA/target axis in thyroid cancer, a type of endocrine system tumor (Table 6).

Thyroid cancer is the most commonly diagnosed endocrine tumor worldwide, with an increasing incidence in the past 20 years 128. To date, a number of miRNAs have been reported to be aberrantly expressed in thyroid cancer and play a vital role in its progression. A study to understand the roles of miR-592 in thyroid cancer found that downregulated miR-592 in thyroid cancer exhibited a short overall survival of patients by promoting cell proliferation, migration, and invasion of thyroid cancer cells. The investigation showed that NEAT1 and neuro-oncological ventral antigen 1 (NOVA1) are targets of miR-592, and the knockdown of NEAT1 and NOVA1 effectively abolish the promotion effects of miR-592 downregulation in thyroid cancer cells, suggesting a vital role of NEAT1/miR-592/NOVA1 axis in thyroid cancer progression 129.

Papillary thyroid cancer (PTC) is the most common form of thyroid cancer, accounting for >80% of thyroid cancer cases. Investigation of the roles of NEAT1 in PTC progression showed that NEAT1 expression was significantly upregulated in PTC tissues and cell lines, and NEAT1 overexpression promoted PTC cell proliferation, invasion, and migration, and inhibited cell apoptosis by increasing the expression level of kallikrein-related peptidase 7 (KLK7) 130 and ATPase family AAA domain-containing protein 2 (ATAD2) 131 via sponging miR-129-5p and miR-106b-5p, respectively.

## NEAT1 role in the tumorigenesis in mobility system tumors

In this section, we summarize and discuss the role of the NEAT1/miRNA/target axis in osteosarcoma, a type of mobility system tumor (Table 6).

Osteosarcoma (OS) is the most common primary malignant bone tumor in children and teenagers. Somatic mutations and epigenetic mechanisms contribute to the progression of OS, such as aberrant activation of oncogenes and dysregulation of ncRNAs 132. Several studies on the role of NEAT1 in OS progression showed that upregulation of NEAT1 in osteosarcoma tissues promoted OS cell proliferation, migration, and invasion, EMT, and inhibited cell apoptosis. Investigation of the mechanism revealed that NEAT1 acts as a ceRNA to regulate the expression of TGF-β1 133, human hypoxia-inducible factor 1α (HIF-1α) 134, and homeobox A13 (HOXA13) 135 by sponging miR-339-5p, miR-186-5p, and miR-34a-5p.

## NEAT1 role in the tumorigenesis in urinary system tumors

In this section, we summarize and discuss the role of the NEAT1/miRNA/target axis in urinary system tumors, including bladder cancer and renal cell carcinoma (Table 6).

### Bladder cancer

Bladder cancer is a common urological malignant tumor in men worldwide and is characterized by a high rate of early systemic dissemination and nearly 170,000 deaths annually 136. Shan et al. 137 revealed that the upregulation of NEAT1 in bladder cancer promotes bladder cancer cell proliferation and inhibits cell apoptosis and cell arrest by sponging miR-410, thereby upregulating the expression of high mobility group box 1 (HMGB1), an accelerator for tumor progression by its immune protective and suppressive functions 138.

### Renal cell carcinoma

Renal cell carcinoma (RCC) is the most common type of kidney cancer and accounts for nearly 95% of all kidney cancer diagnoses 42. In a study to determine the role of NEAT1 in RCC progression, Liu et al. 139 found that NEAT1 expression is upregulated in RCC tissue and cell lines, and high NEAT1 expression is correlated with poor prognosis. Further investigation revealed that NEAT1 enhanced RCC cell proliferation, migration, invasion, and EMT, and inhibited cell cycle progression by sponging miR-34a, thus increasing the expression level of c-Met, a potential therapeutic target in cancers 140.

## NEAT1 in cancer therapy

Conventional treatments for cancer include surgery, chemotherapy, and radiotherapy. However, there is a subset of cancer patients that exhibit metastases and are unresponsive to chemotherapy or radiotherapy owing to tumor heterogeneity, tumor microenvironment, and dysfunction of therapeutic resistance-related genes 141-143. To date, dozens of studies have reported an association between NEAT1 and resistance to chemotherapy or radiotherapy in various cancers (Table 7). They found that knockdown of NEAT1 could sensitize cancer cells to radiation or chemical drugs through NEAT1-mediated ceRNA networks. Therefore, targeting the feedback loop of NEAT1/miRNA/target may be a potential pathway to overcome therapeutic resistance in cancer.

## Conclusions

**Figure 1:**
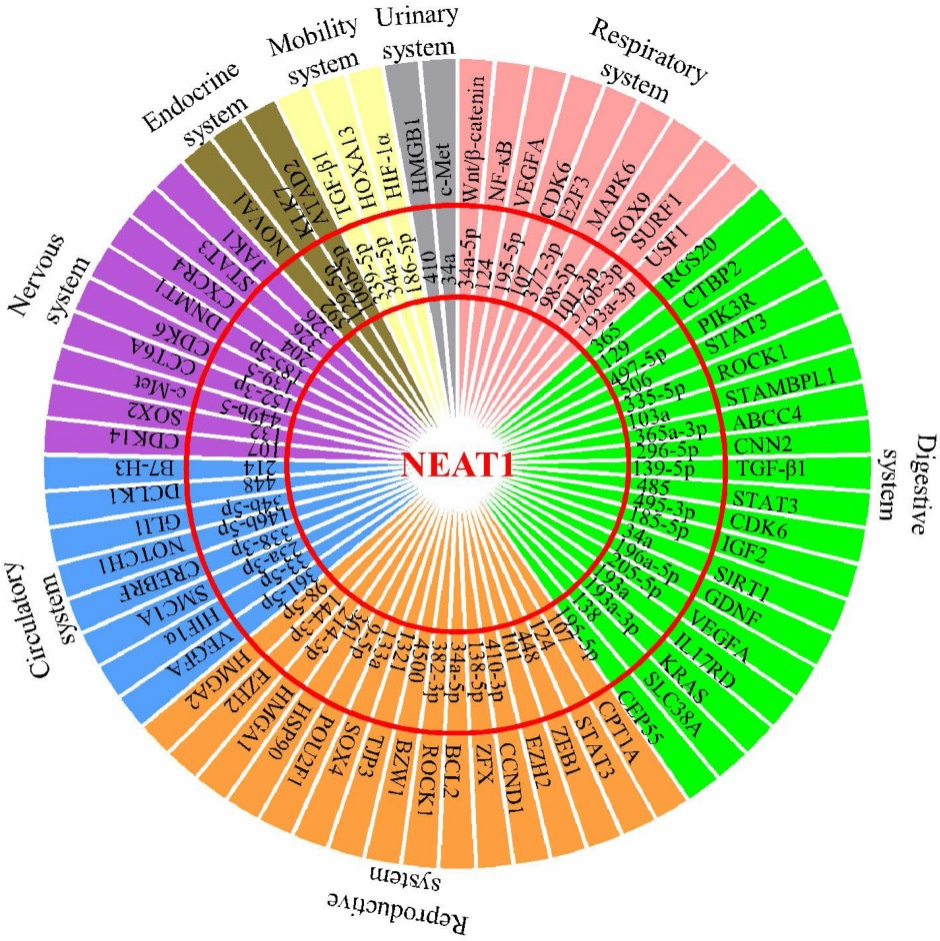
Schematic model shows NEAT1's role as a ceRNA to influence the miRNA environment during development of multiple physiological system tumors.

The effect of malignant cancers is devastating across all physiological systems. The interplay between uncontrolled cancer cell growth, migration, invasion, and inhibition of apoptosis results in inevitable metastasis affecting all organ systems. To date, lncRNA NEAT1 has been reported to be aberrantly expressed in different types of cancers. This review extensively summarized all existing information available on NEAT1's contribution in their development, as NEAT1's role as a ceRNA influences the miRNA environment during tumorigenesis (Figure 1). NEAT1 knockdown studies have revealed a therapeutic potential by redirecting the feedback loop between NEAT1/miRNA/target, thereby increasing efficacy of radio- and chemotherapy. It therefore highlights that NEAT1 is of relevant research interest and its role in therapeutic knockdown to enhance cancer therapies should be considered. However, as a nuclear enriched lncRNA, it should be clarified how NEAT1 sponges so many miRNAs to regulate expression of tumorigenesis-related genes, and whether NEAT1 in the peripheral blood could act as a biomarker for the diagnosis of cancers. In addition, more studies are needed in the future to characterize the role of NEAT1 in tumor microenvironments, such as whether NEAT1 affects the function of tumor infiltrating lymphocytes (TILs), and whether NEAT1 could function as a “messenger lncRNA” for the communication between tumor cells and these immune cells. Overall, this review summarizes and discusses the roles of the NEAT1-miRNA-target axis in the progression of various cancers and provides insight into its potential clinical utility in cancer treatment.

## Figures and Tables

**Table 1:** Roles of NEAT1/miRNA/target axis in respiratory system tumors

Cancer type	MiRNA	Target	Role	Reference
NPC	34a-5p	Wnt/β-catenin	Promoting NPC cell proliferation, migration, invasion, and EMT	21
	124	NF-κB	Promoting NPC cell proliferation and inhibiting cell apoptosis	22
SNSCC	195-5p	VEGFA	Enhancing SNSCC cell viability and inhibiting SNSCC cell apoptosis	25
LSCC	107	CDK6	Promoting LSCC cells proliferation and invasion, and inhibiting LSCC cells apoptosis and cell cycle arrest at G1 phase	28
NSCLC	377-3p	E2F3	Promoting NSCLC cells growth and metastasis	30
	98-5p	MAPK6	Promoting NSCLC cell growth, migration, and invasion	31
	101-3p	SOX9	Promoting NSCLC cell proliferation, migration and invasion	32
	376b-3p	SURF1	Promoting NSCLC cell proliferation, migration, and invasion and inhibiting cell apoptosis	33
LUAD	193a-3p	USF1	Promoting LUAD cell proliferation, invasion, and migration and inhibiting cell apoptosis	40

**Table 2:** Roles of NEAT1/miRNA/target axis in digestive system tumors

Cancer type	MiRNA	Target	Role	Reference
OSCC	365	RGS20	Promoting OSCC cells proliferation and invasion, and inhibiting cell cycle arrest at the G0/G1 phase and apoptosis	45
ESCC	129	CTBP2	Promoting ESCC cell viability and invasion	47
GC	497-5p	PIK3R	Promoting GC cells proliferation, and inhibited GC cells apoptosis	54
	506	STAT3	Promoting GC cells proliferation, migration and invasion	53
	335-5p	ROCK1		55
	103a	STAMBPL1		56
	365a-3p	ABCC4	Promoting GC cells proliferation, colony formation, invasion, and cell cycle	57
HCC	296-5p	CNN2	Promoting HCC cells growth, migration, and invasion	60
	139-5p	TGF-β1		61
	485	STAT3		62
CRC	495-3p	CDK6	Promoting colon cancer cell proliferation, cell cycle, cell migration, and invasion and inhibiting cell apoptosis	65
	185-5p	IGF2	Promoting colon cancer cell migration and invasion	66
	34a	SIRT1	Promoting CRC cells proliferation, migration, and invasion	67
	196a-5p	GDNF		68
	205-5p	VEGFA		69
	193a	IL17RD	Promoting CRC cells proliferation, migration, and invasion, and inhibiting CRC cells apoptosis	70
	193a-3p	KRAS		72
	138	SLC38A		71
	195-5p	CEP55		73

**Table 3:** Roles of NEAT1/miRNA/target axis in reproductive system tumors

Cancer type	MiRNA	Target	Role	Reference
BC	107	CPT1A	Promoting BC cells proliferation, migration, invasion and cell cycle	75
	124	STAT3		76
	448	ZEB1		77
	101	EZH2		78
	410-3p	CCND1	Promoting BC cells proliferation, migration, invasion, and EMT	79
	138-5p	ZFX	Promoting BC cells proliferation, migration, invasion, and inhibiting apoptosis	80
OC	34a-5p	BCL2	Promoting OC cells proliferation and inhibited apoptosis	82
	382-3p	ROCK1	Promoting OC cells metastasis	83
	4500	BZW1	Promoting OC cells proliferation, migration, invasion, and inhibiting apoptosis	84
	1321	TJP3	Promoting OC cells proliferation, migration, invasion, and EMT	85
CC	133a	SOX4	Promoting CC cells proliferation, migration, invasion, and inhibiting apoptosis	88
	9-5p	POU2F1	Promoting CC cells proliferation and migration	87
	361	HSP90	Promoting CC cells proliferation, migration, and EMT	90
EC	214-3p	HMGA1	Promoting EC cells proliferation, migration and invasion	93
	144-3p	EZH2	Promoting EC cells proliferation, migration and invasion	94
PCa	98-5p	HMGA2	Promoting PCa cells proliferation and invasion	96

**Table 4:** Roles of NEAT1/miRNA/target axis in circulatory system tumors

Cancer type	MiRNA	Target	Role	Reference
HA	361-5p	VEGFA	Promoting HemECs proliferation and migration, and inhibiting HemECs apoptosis	100
	33-5p	HIF1α	Promoting HemECs proliferation, migration, and invasion	98
AML	23a-3p	SMC1A	Inhibiting AML cells proliferation, decreasing the number of cells in the G2/M phase, and inducing cell apoptosis	103
	338-3p	CREBRF	Inhibiting AML cells proliferation, migration and invasion, and enhancing AML cells apoptosis	104
T-ALL	146b-5p	NOTCH1	Promoting T-ALL cells proliferation	107
DLBCL	34b-5p	GLI1	Promoting DLBCL cells proliferation, and inhibiting DLBCL cells apoptosis	109
HL	448	DCLK1	Promoting HL cells proliferation and invasion	113
MM	214	B7-H3	Promoting M2 macrophage polarization	116

**Table 5:** Roles of NEAT1/miRNA/target axis in nervous system tumors

Cancer type	MiRNA	Target	Role	Reference
Glioma	107	CDK14	Promoting glioma cells proliferation, migration, and invasion	118
	132	SOX2		119
	449b-5	c-Met		120
	152-3p	CCT6A	Promoting glioma cells proliferation, migration, and invasion, inhibiting cells apoptosis	121
	139-5p	CDK6		122
	185-5p	DNMT1	Promoting glioma cells proliferation, migration, invasion, and EMT, inhibiting cells apoptosis	123
RB	204	CXCR4	Promoting RB cells proliferation and migration, and inhibiting cells apoptosis	125
NB	326	JAK1, STAT3	Promoting NB cells proliferation, and inhibiting cells apoptosis	127

**Table 6:** Roles of NEAT1/miRNA/target axis in endocrine system, mobility system, and urinary system tumors

System	Cancer type	miRNA	Target	Role	Reference
Endocrine system	Thyroid carcinoma	592	NOVA1	Promoting thyroid cancer cells proliferation, migration, and invasion	129
	PTC	129-5p	KLK7	Promoting PTC cells proliferation, migration, and invasion, and inhibiting cells apoptosis	130
		106b-5p	ATAD2		131
Mobility system	OS	339-5p	TGF-β1	Promoting OS cells proliferation, migration, and invasion	133
		34a-5p	HOXA13	Promoting OS cells proliferation and inhibiting cells apoptosis	135
		186-5p	HIF-1α	Promoting OS cells proliferation, invasion, and EMT	134
Urinary system	Bladder cancer	410	HMGB1	Promoting bladder cancer cells proliferation and inhibiting cell apoptosis and cell arrest	137
	RCC	34a	c-Met	Promoting RCC cells proliferation, migration, invasion,and EMT, and inhibiting cell cycle progression	140

**Table 7:** Roles of NEAT1/miRNA/target axis in therapeutic resistance of cancers

Cancer type	MiRNA	Target	Chemical-/radio- resistance	Reference
BC	211	HMGA2	5-fluorouracil (5-FU)	144
CRC	150-5p	CPSF4		145
OC	770-5p	PARP1	Cisplatin (CDDP)	146
ATC	9-5p	SPAG9		147
OS	34c	BCL-2		148
		CCND1		
PCA	204-5p	ACSL4	Docetaxel	149
	34a-5p			
Bladder cancer	214-3p	Wnt/β-catenin	Doxorubicin (DOX)	150
OC	194	ZEB1	Paclitaxel (PTX)	151
EC	361	STAT3		152
HCC	204	ATG3	Sorafenib	153
	335	c-Met		154
RCC	34a	c-Met		140
NPC	129	Bcl-2	Suberoylanilide hydroxamic acid (SAHA)	155
NPC	204	ZEB1	Radiation	156
HCC	101-3p	WEE1		157
CC	193b-3p	CCND1		158

## Abbreviations

NEAT1: nuclear paraspeckle assembly transcript 1
LncRNA: Long non-coding RNA
CeRNA: competing endogenous RNA
MiRNAs: MicroRNAs
PSPC1: Paraspeckle component 1
SFPQ: Splicing factor proline/glutamine rich
P54nrb: 54 kDa nuclear RNA- and DNA-binding protein
AD: Alzheimer's disease
EMT: Epithelial-mesenchymal transition
NPC: Nasopharyngeal carcinoma
SNSCC: Sinonasal squamous cell carcinoma
VEGFA: Vascular endothelial growth factor A
LSCC: Laryngeal squamous cell carcinoma
CDK6: Cyclin-dependent kinase 6
NSCLC: Non-small cell lung cancer
MAPK6: Mitogen-activated protein kinase 6
LUAD: Lung adenocarcinoma
USF1: Upstream stimulating factor 1
OSCC: Oral squamous cell carcinoma
HNSCC: Head and neck squamous cell carcinoma
TNM: Tumor size, Node location, and Metastasis
RGS20: G protein signaling 20
ESCC: Esophageal squamous cell carcinoma
CTBP2: C-terminal-binding protein 2
GC: Gastric cancer
STAT3: Signal transducer and activator of transcription 3
PIK3R1: Phosphoinositide-3-kinase regulatory subunit 1
ROCK1: Rho associated coiled-coil containing protein kinase 1
STAMBPL1: STAM binding protein like 1
ABCC4: ATP binding cassette subfamily C member 4
HCC: Hepatocellular carcinoma
CNN2: Calponin 2
TGF-β1: Transforming growth factor-β1
CRC: Colorectal cancer
IGF2: Insulin-like growth factor 2
SIRT1: Sirtuin-1
GDNF: Glial cell-derived neurotrophic factor
IL17RD: Interleukin 17 receptor D
CEP55: Centrosomal protein 55
BC: Breast cancer
ZEB1: Zinc finger E-box binding homeobox 1
EZH2: Enhancer of zeste homolog 2
CCND1: Cyclin D1
ZFX: zinc finger protein X-linked
OC: Ovarian cancer
BCL2: B-cell lymphoma-2
BZW1: Basic leucine zipper and W2 domain‑containing protein 1
TJP3: Tight junction protein 3
CC: Cervical cancer
POU2F1: POU class 2 homeobox 1
SOX4: SRY-box transcription factor 4
HSP90s: 90-kDa heat shock proteins
EC: Endometrial carcinoma
HMGA1: High mobility group AT-hook 1
PCa: Prostate cancer
HMGA2: High mobility group AT-hook 2
HA: Hemangioma
HemECs: Hemangioma endothelial cells
AML: Acute myeloid leukemia
SMC1A: Structural maintenance of chromosomes 1A
CREBRF: CREB3 regulatory factor
T-ALL: T-cell acute lymphoblastic leukemia
DLBCL: Diffuse large B-cell lymphoma
GLI1: GLI family zinc finger 1
HL: Hodgkin's lymphoma
DCLK1: Doublecortin-like kinase 1
MM: Multiple myeloma
CDK14: Cyclin-dependent kinase 14
SOX2: SRY-box transcription factor 2
CCT6A: Chaperonin containing the TCP1 subunit 6A
DNMT1: DNA methyltransferase 1
RB: Retinoblastoma
CXCR4: C-X-C chemokine receptor type 4
NB: Neuroblastoma
JAK1: Janus kinase 1
NOVA1: Neuro‑oncological ventral antigen 1
PTC: Papillary thyroid cancer
KLK7: Kallikrein-related peptidase 7
ATAD2: ATPase family AAA domain-containing protein 2
OS: Osteosarcoma
HIF-1α: Hypoxia-inducible factor 1α
HOXA13: Homeobox A13
HMGB1: High mobility group box 1
RCC: Renal cell carcinoma
TILs: tumor infiltrating lymphocytes
